# A Case of Lisinopril‐Induced Hallucinations

**DOI:** 10.1002/jgf2.70099

**Published:** 2026-03-23

**Authors:** Jack Golder, Julia Isom

**Affiliations:** ^1^ Edward via College of Osteopathic Medicine—Carolinas Spartanburg South Carolina USA; ^2^ Roper St Francis Healthcare Charleston South Carolina USA

**Keywords:** ACE inhibitor, adverse drug reaction, elderly, hallucinations, lisinopril

## Abstract

**Background:**

Lisinopril is generally well tolerated, but rare neuropsychiatric adverse effects have been reported.

**Case:**

We describe an 89‐year‐old woman who developed vivid nocturnal auditory and visual hallucinations approximately 3 months after starting lisinopril for hypertension. Neurologic examination, imaging, and laboratory evaluation were unremarkable. Hallucinations resolved completely within 2 weeks of drug discontinuation, with no recurrence on follow‐up.

**Conclusion:**

Older adults may be particularly susceptible to medication‐induced neuropsychiatric effects. Recognition of lisinopril as a reversible cause of hallucinations is essential to avoid misdiagnosis and unnecessary treatment.

## 1 Introduction

Lisinopril, marketed under the brand names *Prinivil*, *Qbrelis*, and *Zestril*, is an angiotensin‐converting enzyme (ACE) inhibitor prescribed for the management of hypertension, heart failure, and certain forms of kidney disease, owing to its effects on vascular tone and renal homeostasis [1].

The antihypertensive and heart failure benefits of lisinopril are primarily due to suppression of the renin‐angiotensin‐aldosterone system. The mechanism of action of lisinopril is inhibition of ACE, an enzyme responsible for converting angiotensin I to angiotensin II. By blocking ACE, lisinopril decreases plasma angiotensin II levels, leading to reduced vasopressor activity and decreased aldosterone secretion. This results in vasodilation and the lowering of blood pressure [2, 3, 4].

Overall, the risk profile of ACE inhibitors is well characterized, with most adverse effects being mild and reversible. The most common side effects include headache, dizziness, and cough in patients treated for hypertension [2, 3, 4]. Hyperkalemia can also occur as well as minor increases in serum creatinine and blood urea nitrogen. Additional reported effects can include angioedema, pancreatitis, pruritus, and, rarely, skin reactions such as Stevens‐Johnson syndrome and toxic epidermal necrolysis [5].

Hallucinations induced by lisinopril are a rare but documented adverse effect. The mechanism is not fully elucidated, but it is suggested that this is due to an idiosyncratic neuropsychiatric reaction to ACE inhibition. Proposed mechanisms include altered central nervous system neurotransmitter balance due to changes in the renin‐angiotensin system, which is present in the brain and may influence dopaminergic and cholinergic pathways [6].

With the rarity of the presentation, it is imperative to review the diagnostic considerations for acute onset hallucinations in elderly individuals. This case report details the clinical presentation, diagnostic evaluation, and management strategies for ACE inhibitor induced hallucinations.

## 2 Case Report

An 89‐year‐old female presented to her primary care provider with a 2 month history of nocturnal auditory and visual hallucinations. The patient reported perceiving that a movie was being filmed outside her residence between approximately 12:00 a.m. and 6:00 a.m. She described seeing numerous parked cars, film crews, bright lights, and hearing conversations about filming “giants and little people.” The patient lived alone, and her neighbors denied observing any such events. She denied experiencing hallucinations during the daytime. Regarding her sleep habits, she reported going to bed around 11:00 p.m. nightly and denied difficulty with sleep initiation or maintenance.

Her past medical history was notable for anxiety, arthritis, hyperlipidemia, gastroesophageal reflux disease, uncontrolled hypertension, chronic gastritis, prediabetes, and a prior myocardial infarction. Thirteen days before presentation, she had been evaluated in the emergency department for a urinary tract infection and was subsequently hospitalized overnight for nausea and vomiting secondary to trimethoprim‐sulfamethoxazole therapy. She denied any history of dementia, cognitive decline, or prior hallucinations and reported adherence to all prescribed medications.

Vital signs were within normal limits except for elevated blood pressure (140/68 mmHg). On examination, she was alert and oriented to person, place, and time, with no focal neurological deficits or other significant physical findings. Review of prior hospitalization records revealed an unremarkable head computed tomography scan. Laboratory results from that admission were notable for mild hyponatremia (131 mEq/L; reference range 135–145 mEq/L) and hypokalemia (3.1 mEq/L; reference range 3.5–5.0 mEq/L), both of which had been corrected prior to discharge.

Medication review revealed the patient was taking ondansetron, amlodipine, polyethylene glycol, metoprolol, famotidine, omeprazole, and lisinopril, the latter initiated approximately 3 months prior. She denied alcohol consumption and any history of illicit drug use. Given the temporal association between symptom onset and lisinopril initiation, a plan of care was established that included discontinuation of lisinopril and further laboratory evaluation, including serum folate, vitamin B12, rapid plasma reagin, and a basic metabolic panel. She was instructed to follow up in 2 weeks for reassessment.

At the follow‐up visit, the patient reported full adherence to the recommended plan, including discontinuation of lisinopril. She denied any recurrence of auditory or visual hallucinations. Repeat laboratory testing revealed no abnormalities. Based on these findings, lisinopril was permanently discontinued, and her metoprolol dosage was increased for blood pressure management. The patient has since remained free of hallucinations.

## 3 Discussion

In the evaluation of acute hallucinations in an outpatient setting, a detailed history should be elicited, including onset, duration, frequency, sensory modality, associated symptoms, and impact on daily functioning [7, 8]. The provider should also review psychiatric, neurologic, medical, and medication history, with particular attention to recent medication changes, substance use, and withdrawal states [8, 9]. Collateral information from family or caregivers is essential to clarify the course and context of symptoms, especially in older adults [8].

A comprehensive physical examination, including vital signs, is required to identify systemic illness or toxicity. In addition, a detailed neurologic and mental status assessment should be performed to evaluate for focal deficits, cognitive impairment, and signs of delirium. Assessment for delirium is particularly important in older adults and those with acute onset of symptoms [7, 8, 9].

When formulating a differential diagnosis, it is important to consider primary psychiatric disorders (e.g., schizophrenia, mood disorders with psychotic features), underlying medical conditions (e.g., delirium, neurodegenerative diseases, metabolic abnormalities), and substance‐induced etiologies, as well as benign phenomena such as hypnagogic or hypnopompic hallucinations [8, 9].

Clinically, hallucinations associated with lisinopril are most often visual, but auditory or mixed types have also been reported. They typically occur in older adults, particularly those with pre‐existing cognitive impairment or dementia and may present within days to weeks of drug initiation [6]. Other neuropsychiatric features can include confusion, memory impairment, and mood alterations [10].

Hallucinations typically resolve within days to weeks following discontinuation of lisinopril. The primary management approach involves prompt cessation of the medication and initiation of an alternative antihypertensive agent as appropriate [6, 10].

The acute onset of hallucinations in older adults warrants prompt and thorough evaluation to identify reversible causes. A comprehensive medication review is essential, as adverse drug reactions may present atypically in this population. Although exceedingly rare, this case highlights lisinopril as a potential cause of hallucinations. Clinicians should maintain a high index of suspicion for medication‐induced neuropsychiatric effects to ensure timely recognition, discontinuation of the offending agent, and resolution of symptoms.

## Author Contributions

**Jack Golder:** conceptualization, formal analysis, investigation, methodology, writing – original draft, writing – review and editing. **Julia Isom:** conceptualization, data curation, formal analysis, investigation, methodology, project administration, supervision, validation, visualization, writing – review and editing.

## Consent

Consent including a signed release from the patient was obtained prior to research initiation.

## Conflicts of Interest

The authors declare no conflicts of interest.

## Data Availability

Data sharing not applicable to this article as no datasets were generated or analysed during the current study.
